# Free and universal, but unequal utilization of primary health care in the rural and urban areas of Mongolia

**DOI:** 10.1186/s12939-017-0572-4

**Published:** 2017-05-08

**Authors:** Javkhlanbayar Dorjdagva, Enkhjargal Batbaatar, Mikael Svensson, Bayarsaikhan Dorjsuren, Burenjargal Batmunkh, Jussi Kauhanen

**Affiliations:** 1 Department of Health Policy and Management, School of Public Health, Mongolian National University of Medical Sciences, Zorig street, Ulaanbaatar, 14210 Mongolia; 2 Institute of Public Health and Clinical Nutrition, Faculty of Health Sciences, University of Eastern Finland, Kuopio, Finland; 3 Health Metrics Unit, the Sahlgrenska Academy, University of Gothenburg, Gothenburg, Sweden; 4 Department of Health Systems Governance and Financing, WHO, Geneva, Switzerland; 5 Department of Physiology, School of Pharmacy and Bio Medicine, Mongolian National University of Medical Sciences, Ulaanbaatar, Mongolia

**Keywords:** Primary health care, Inequality, Horizontal inequity, Urban and rural disparity, Mongolia

## Abstract

**Background:**

The entire population of Mongolia has free access to primary health care, which is fully funded by the government. It is provided by family health centers in urban settings. In rural areas, it is included in outpatient and inpatient services offered by *rural soum* (district) health centers. However, primary health care utilization differs across population groups. The aim of this study was to evaluate income-related inequality in primary health care utilization in the urban and rural areas of Mongolia.

**Methods:**

Data from the Household Socio-Economic Survey 2012 were used in this study. The Erreygers concentration index was employed to assess inequality in primary health care utilization in both urban and rural areas. The indirect standardization method was applied to measure the degree of horizontal inequity.

**Results:**

The concentration index for primary health care at family health centers in urban areas was significantly negative (−0.0069), indicating that utilization was concentrated among the poor. The concentration index for inpatient care utilization at the *soum* health centers was significantly positive (0.0127), indicating that, in rural areas, higher income groups were more likely to use inpatient services at the *soum* health centers.

**Conclusions:**

Income-related inequality in primary health care utilization exists in Mongolia and the pattern differs across geographical areas. Significant pro-poor inequality observed in urban family health centers indicates that their more effective gatekeeping role is necessary. Eliminating financial and non-financial access barriers for the poor and higher need groups in rural areas would make a key contribution to reducing pro-rich inequality in inpatient care utilization at *soum* health centers.

## Background

WHO declared in 1978 that ensuring primary health care (PHC) is a key strategy for reaching the goal of “health for all by the 2000” – guaranteeing health equity by virtue of its accessible and affordable characteristics [1]. The PHC concept refers to “the principles of equity, participation, intersectoral action, appropriate technology and a central role played by the health system” [2].

The World Health Report 2008 strongly reaffirms the importance of a well-organized PHC model in creating a more equitable health system and moving toward universal health coverage (UHC) – everyone able to receive comprehensive and quality health services at the right time without any financial hardship [3].

Mongolia has a population of only three million [4]; however, it is the nineteenth largest and the most sparsely populated country in the world. Providing social services, particularly in the countryside, is no easy task.

Before the 1990s, during the socialist era, the country had a centralized health system whereby the government was responsible for both financing and providing services [5]. Under this scheme, everyone was ensured free access to full coverage.

Despite improvements in population health, there were issues in the system, including inefficiency at hospital sector, lack of responsiveness to patient rights and backward PHC [5, 6].

Polyclinics were responsible for PHC in urban areas. They were legally autonomous units. There were different polyclinics for children, men and women. Each one provided health services in its area by means of a laboratory, medical equipment, physicians and specialists [7].

Polyclinics in rural areas were associated with specific hospitals. At the lower level of rural areas, small hospitals or standalone primary care units without beds provided PHC, whereas feldshers did so at the subdistrict (village) level [7].

Mongolian public finances were highly dependent on subsidies from the U.S.S.R. (the Union of Soviet Socialist Republics) (accounting for around 30% of Mongolian GDP). The collapse of the Soviet Union and transition from a centrally planned to market economy had drastic budgetary consequences [8]. A financial crisis depleted funds for the public sector, including health services [7].

After the transition to a market economy, the government adopted a number of reforms to lighten the burden of public finances and ensure more equitable and higher quality health services. One of the main reforms was structuring PHC. By the late 1990s, the concept of PHC had become a key priority for health policies. With the assistance of the Asian Development Bank (ADB), the goal was not only to improve access to quality essential health services and ensure financial sustainability of the health system, but improve the quality of health services [6]. By early 2002, PHC in urban areas, including the Ulaanbaatar (capital city) and Aimag (provincial) centers, had fully transitioned to family group practices (FGPs) [5]. There is currently universal access to PHC, which is fully funded by the government budget [5, 9]. However, there are substantial differences when it comes to PHC services in urban and rural areas with regard to setting, type of provider and function [5].

### PHC in urban areas

According to the 2011 Health Act, FGP was renamed Family Health Centers (FHCs), which provide PHC in urban areas [5]. FHCs are private organizations fully funded by the government budget based on a risk-adjusted capitation payment method. A typical FHC has 3–4 doctors, and the average nurse-to-doctor ratio is 1 [5]. In 2011, 6% of the public healthcare expenditure was allocated to FHCs [10]. In 2014, a total of 218 FHCs provided PHC services for approximately 2 million individuals in urban areas [11]. As a result of migration from rural to urban areas, the workload of FHCs has risen dramatically over the past decade [12, 13].

### PHC in rural areas

PHC services in rural areas are provided by *soum* (district) health centers, which are owned by local governments and also funded according to the risk-adjusted capitation method. They have doctors, nurses, midwives, feldshers, etc. [5]. Due to the distance to the upper level hospitals, each center also provides inpatient care with 5–15 beds for the rural population.

In 2014, there were 271 *soum* health centers and 19 village (subdistrict) health centers that provided services for their areas [11]. About 20.8% of public healthcare expenditure was on *soum* health centers [10]. This paper assesses inequality in utilization of inpatient and outpatient care at the centers.

Despite government reforms, the healthcare sector has faced a number of problems, including inefficiency, high out-of-pocket share of total expenditures and the persistence of socioeconomic inequality [14–17]. A wealth of evidence suggested that the problems are linked to the capacity of PHC and a weak gatekeeping system at this level. Thus, strengthening PHC is a high priority for health policy reform [14–16].

One concern is socioeconomic inequality in PHC utilization; however, few studies have specifically addressed the issue.

Our previous study analyzed the degree of income-related inequality in healthcare utilization at the primary, secondary and tertiary levels. The results showed that pro-poor inequality in PHC utilization tended to increase between 2008 and 2012. Such inequality stemmed mainly from location, educational level and income [15]. Nevertheless, we are not aware of any data about how inequality in PHC utilization differs across geographical areas.

The current study attempts to analyze the degree of inequality in PHC services in the urban and rural areas of Mongolia. The aim of this paper is to evaluate income-related inequality in PHC utilization in those areas.

## Methods

### Data

Our data were obtained from the Mongolian Household Socio-Economic Survey (HSES) 2012. The HSES is a nationally representative cross-sectional study. The National Statistical Office conducts the HSES every year to “evaluate and monitor the income and expenditure of households, to provide the basis for the poverty monitoring system, poverty mapping and poverty reduction policies, to update the basket and the weights for the consumer price index, and to offer inputs to the national accounts” [18]. The survey consists of a wide range of questions concerning demographics, socioeconomic indicators, welfare payments, health status, housing, educational level, etc. The HSES 2012 included 12,811 households consisting of 47,908 individuals.

The main criterion for inclusion in this study was that individuals be 18 or older. The following respondents were also excluded: a) heads of households or students who had been away from home for the past 11 months or longer; b) anyone else who had been away from home for the past 6 months or longer. After applying the inclusion criteria, we eliminated 20 cases with missing data. As a result, 30,547 respondents were involved in the study.

### Measuring healthcare utilization

Based on the data availability, we generated three healthcare utilization variables, all of which are binary. Measurements of FHC and *soum* health center utilization in urban and rural areas were based on whether respondents had received outpatient care at any FHCs and *soum* health centers over the past month. Inpatient care utilization at the *soum* health centers was measured by whether respondents had been hospitalized over the past 12 months.

### Measuring socioeconomic status

This study used household income as a socioeconomic indicator. First, we estimated household net income over the past year from all sources (salaries, self-employment, agricultural income, private sources, pension, etc.). Second, we calculated household income per equivalent adult using the OECD modified equivalence scale: “1 for the head of the household, 0.5 for each additional adult and 0.3 for each child.”

### Need variables

Age, gender and self-reported health were used as need variables. We generated 14 dummy variables in accordance with age and gender (females age 18–24, 25–34, 35–44, 45–54, 55–64, 65–74, and 75 or older; males age 18–24, 25–34, 35–44, 45–54, 55–64, 65–74, and 75 or older). The reference group consisted of females age 18–24.

It is well-documented that self-reported health is a potential predictor of subsequent mortality [19, 20]. For that reason, we used three self-reported health variables. The respondents were asked: a) Do you have any disabilities? b) Have you had any health complaints over the past month? c) Have you missed work, school or daily activities due to illness over the past month (number of days)?

### Other independent variables

Non-need variables are marital status, economic activity, educational level, household size and distance to the nearest healthcare center. Marital status include married/living together (the reference group), divorced/separated, widowed and single/never married. Four types of economic status were included: employed (baseline), herder, self-employed, inactive and unemployed. Educational level was lower or none (ISCED 0 to 1), lower secondary (ISCED 2), upper secondary (ISCED 3 to 4) and postsecondary (ISCED 5 to 6). Household size and distance to the nearest healthcare center are continuous variables. Since everyone in Mongolia has free access to PHC, insurance status was not considered.

### Statistical analysis

The statistical analysis consisted of four steps to assess income-related inequality in PHC utilization in urban and rural areas: (a) we estimated the determinants of PHC utilization based on a linear regression model; (b) we estimated income-related inequality in PHC utilization based on a concentration index; (c) we calculated the degree of inequity in PHC utilization; (d) we decomposed the contribution of each determinant to overall inequality.

### Measuring inequality in healthcare utilization

We used the concentration index that had been developed in health economics field as a standard tool for measuring socioeconomic inequality in health and health care [21]. The index shows the covariance of PHC utilization and the fractional rank of income distribution as:

1 $$ C I = \frac{2}{\mu}\  c o{v}_w\left({y}_i,\ {R}_i\right) $$

where *i* is an individual, *y*_*i*_ is healthcare utilization, *μ* is the mean healthcare utilization (*y*) and *R*_*i*_ is the fractional income rank of individual *i*. The index ranges between −1 and +1. A negative value indicates that healthcare utilization is concentrated among the poor, and a positive value indicates that it is concentrated high among the rich. An index of 0 indicates that there is no socioeconomic inequality in healthcare utilization [21]. However, the index has a limitation when a healthcare utilization variable is binary. The index shrinks as the mean increases [22]. Several normalized concentration indices have been proposed. Wagstaff’s and Erreyger’s (EI) indices have been widely applied. We used EI, which is formulated as [23]:

2 $$ E(h) = \frac{4\upmu}{\left({b}_n-{a}_n\right)}\  C(h) $$

where *C(h)* is the standard concentration index, which is presented in Equation 1. The *μ* is mean healthcare utilization in a population. *b*_*n*_ and *a*_*n*_ are the upper and lower bounds of healthcare utilization.

### Measuring horizontal inequity in health care utilization

Horizontal inequity (HI) in healthcare utilization is avoidable inequality. This was estimated by subtracting the concentration index of need-standardized from the concentration index. We applied an indirect standardization method. When a dependent variable is binary, a nonlinear regression model is preferable. Nonetheless, we used ordinary least square (OLS) regression for both indirect standardization and decomposition analysis. Similar studies have concluded that linear and nonlinear models produce similar results [24]. Moreover, an approximation error was found in the decomposition analysis with nonlinear models [25].

First, coefficients of the OLS for PHC utilization (${h}_i$) were obtained as follows:

3 $$ {h}_i=\alpha + {\displaystyle \sum_j}{\beta}_{j\kern0.5em }{\chi}_{j, i} + {\displaystyle \sum_k}{\gamma}_k{z}_{k, i} + {\varepsilon}_i $$

where ${h}_i$ is PHC utilization by an individual, ${\chi}_j$ represents a set of need variables consisting of age, gender and health needs; ${z}_k$ is a set of non-need variables, including the logarithm of household income per equivalent adult, marital status, economic activity, educational level, household size and distance to the nearest healthcare center; $\alpha, \beta$ and $\gamma$ are the parameter vectors, and ${\varepsilon}_i$ is an error term.

Second, we estimated need-predicted values of PHC (*ĥ*_*i*_^*x*^) based on the parameter vectors ($\alpha, {\beta}_j$, ${\gamma}_{\kappa}$), individual values of the need variables (${\chi}_j$), and sample means of the non-need (*z*_*k*,*i*_) variables from Equation 3. The predicted values were obtained by the formula below:

4 $$ {\widehat{h}}_i^x=\widehat{\alpha} + {\displaystyle \sum_j}{\widehat{\beta}}_j{\chi}_{j, i} + {\displaystyle \sum_k}{\widehat{\gamma}}_k{z}_k^m $$

Third, indirectly standardized PHC utilization (*ĥ*_*i*_^*IS*^) was estimated from the difference between actual (*h*_*i*_) and need-predicted PHC utilization (*ĥ*_*i*_^*X*^), and the sample mean (*h* ^*m*^) was added [21].

5 $$ {\widehat{h}}_i^{IS} = {h}_i - {\widehat{h}}_i^X + {h}^m $$

### Decomposition analysis

In order to identify the determinant that contributes most to income-related inequality in PHC utilization, we applied a decomposition analysis. The linear additive model is known to be more convenient for such an analysis. In addition, some reports have found that are not any differences between the linear and the nonlinear results [21]. Since we calculated in accordance with binary dependent variables, the decomposition of concentration index was multiplied by 4 and $\mu$_h_ to obtain the EI (equation 6).

6 $$ E=4\left[{\displaystyle \sum_j}{\beta}_j{\mu}_{x_j}{C}_{x_j}+{\displaystyle \sum_k}{\gamma}_k{\mu}_{z_k}{C}_{z_k}\right] $$

Where *μ* indicates the mean, *β* and $\gamma$ are the coefficients of the *x* and *z*, respectively.

We calculated confidence intervals for HI indices using the bootstrapping method with 1,000 replications. We performed a statistical analysis with the STATA/IC 13.1 (StataCorp, 2013).

## Results

Table 1 shows the results of descriptive statistics for all variables by geographic area. Outpatient care at *soum* health centers in rural areas is generally identical with PHC at FHC in urban areas when it comes to function. The results from a t-test show that adults in rural areas used outpatient care at *soum* health centers/FHC to a significantly greater extent than those in urban areas. According to the survey, approximately 5.97% of rural dwellers used inpatient care at a *soum* health center.

**Table 1:** Descriptive statistics

Variable	Urban *N* = 17,354	Rural *N* = 13,193
Health variables		
Disability^a^	5.2%	5.9%
A number of missed work/school days in the past month, median, min and max	0 (0, 31)	0 (0, 30)
Any health problem in last month a	7.8%	6.7%
Age and gender		
female 18–24^a,b^	10.7%	10.3%
female 25–34	12.7%	12.0%
female 35–44	11.8%	11.5%
female 45–54	9.7%	9.5%
female 55–64	5.0%	4.4%
female 65–74	2.5%	2.2%
female 74<	1.5%	1.5%
male 18–24	9.9%	10.0%
male 25–34	11.3%	12.0%
male 35–44^a^	10.0%	11.1%
male 45–54	8.1%	8.9%
male 55–64	3.8%	3.9%
male 65–74	1.9%	1.8%
male 74<	1.0%	0.9%
Marital status		
Married/living together^a,b^	62.9%	64.3%
Divorced/separated a	4.1%	2.3%
Widowed	8.4%	8.5%
Single/never married	24.5%	24.9%
Employment status		
Employed^a,b^	44.7%	29.1%
Herder^a^	2.0%	33.3%
Self-employed^a^	9.6%	5.1%
Inactive^a^	34.4%	24.0%
Unemployed^a^	9.3%	8.6%
Education level		
Lower or no education^a,b^	6.4%	20.2%
Lower secondary^a^	12.3%	28.0%
Upper secondary^a^	53.8%	40.3%
Postsecondary	27.6%	11.6%
Log income per capita, median, min and max	15.2 (11.9, 21.4)	14.7 (11.9, 18.3)
Household size, median (min, max)	4 (1, 15)	4(1, 14)
Log distance to the nearest healthcare center, median (min, max)	0.0(−4.60, 3.47)	0.18 (−3.91, 5.07)
Health care utilization		
FHC/Outpatient care at the *soum* health center	1.1%	2.7%
Inpatient care at *soum* health center	NA	5.9%

^a^Statistically significant difference (*p* < 0.05) between rural and urban areas

^b^Reference group

The percentage of those who had a disability was significantly higher in rural areas. People in urban areas reported more health problems over the past month. There was a higher percentage of married individuals and a lower percentage of divorced individuals in rural areas.

The percentages of those who were working, self-employed, inactive or unemployed were significantly higher in urban areas, whereas more herders (33%) lived in rural areas.

The urban population tended to have significantly greater incomes and higher educational levels. Longer distances to the nearest healthcare center was reported in rural than urban areas.

Table 2 shows concentration indices and HI indices. The EI for FHC visits is negative and statistically significant (*p* < 0.01), indicating that, in urban areas, lower income groups were more likely to use PHC than those with higher incomes. After controlling for need variables, the HI index became smaller but remained negative. Thus, FHC care was distributed in favor of the poor.

**Table 2:** Inequality and inequity in primary health care utilization in Mongolia

Health care use	EI	HI
FHC (confidence interval)	**−0.0069**	**−0.0057**
	(−0.0104, −0.0034)	(−0.0092, −0.0023)
Outpatient care at the *soum* health center (confidence interval)	0,0023	−0,0035
	(−0.0044, 0.0090)	(−0.0089, 0.0021)
Inpatient care at *soum* health center (confidence interval)	**0,0127**	0,0063
	(0.0027, 0.0227)	(−0.0031, 0.0158)

Significant indices are in bold, at the significance level of 0.05

*EI* denotes Erreygers’ concentration index, *HI* represents horizontal Inequity

For outpatient care utilization at s*oum* health centers, the EI is positive and the HI index is negative; nevertheless, both indices were statistically insignificant (*p* > 0.05). For that reason, the other results of outpatient care utilization at *soum* health centers were omitted from the subsequent analysis.

The EI for inpatient care utilization at *soum* health centers was significantly positive (0.013), indicating that, in rural areas, the rich tended to use inpatient care at *soum* health centers more than the poor. After accounting need, the HI index for inpatient care at *soum* health center is 0.006; however, it was statistically insignificant.

Columns 2 and 5 in Table 3 show the results of the regression analysis. All reference groups, including women age 18–24, married/living together, lower or no education, and employed, were omitted.

**Table 3:** Decomposition of concentration index for FHC and inpatient care at soum health centers in urban and rural areas, Mongolia

Variable	FHC			Inpatient care at s*oum* health centers		
	Regression coefficient	CI	Contribution, %	Regression coefficient	CI	Contribution, %
Disability	**−0.009**	−0.1945	−4.78%	**0.0664**	−0.0259	−2.92%
A number of missed work/school days	**−0.005**	−0.0405	−5.03%	**0.0023**	0.0692	1.99%
Any health problem in last month	**0.160**	−0.0323	22.22%	**0.0963**	0.0658	11.82%
female 25–34	0.001	0.0360	−0.28%	0.0054	−0.0689	−1.45%
female 35–44	−0.001	0.0242	0.20%	0.0026	−0.0300	−0.28%
female 45–54	0.005	0.0035	−0.09%	0.0091	0.0945	2.42%
female 55–64	0.006	−0.0212	0.38%	**0.0431**	0.1503	8.99%
female 65–74	**0.020**	−0.1382	4.03%	**0.0912**	0.0715	4.75%
female 74<	−0.005	−0.1514	−0.68%	**0.0435**	0.0373	0.71%
male 18–24	−0.001	−0.0516	−0.26%	−0.0150	0.0011	−0.05%
male 25–34	−0.002	0.0437	0.49%	**−0.0277**	−0.1018	11.49%
male 35–44	−0.002	0.0611	0.76%	−0.0173	−0.0392	2.38%
male 45–54	−0.002	0.0274	0.27%	**−0.0259**	0.0288	−2.02%
male 55–64	−0.002	−0.0287	−0.11%	**0.0279**	0.2025	6.76%
male 65–74	0.007	−0.0195	0.15%	0.0293	0.1647	2.71%
male 74<	−0.006	−0.1536	−0.53%	**0.0434**	0.2245	2.87%
Log income	−0.001	0.0291	33.25%	0.0018	0.0288	24.36%
Divorced/separated	0.005	−0.1477	1.72%	0.0080	−0.1809	−1.04%
Widowed	0.001	−0.1448	0.80%	**0.0180**	−0.0083	−0.41%
Single/never married	0.002	−0.0524	1.27%	**−0.0258**	−0.0071	1.39%
Herder	0.008	−0.3677	2.35%	−0.0057	−0.1343	9.48%
Self-employed	−0.001	0.4320	3.51%	**−0.0209**	0.3812	−11.17%
Inactive	0.000	−0.1388	0.71%	−0.0019	0.0148	−0.19%
Unemployed	0.000	−0.3676	−0.59%	−0.0137	−0.2884	9.74%
Lower secondary	**−0.009**	−0.3230	−35.21%	**−0.0183**	−0.1246	20.81%
Upper secondary	**−0.012**	−0.0473	0.67%	**−0.0225**	0.0873	−24.32%
Third-level education	**−0.015**	0.2946	72.68%	**−0.0178**	0.4338	−24.81%
Household size	0.000	−0.0275	−0.81%	−0.0016	−0.0223	4.51%
Log distance to the nearest health center	−0.001	0.5650	−6.55%	**−0.0037**	−0.1437	20.74%

Significant regression coefficients are in bold, at the significance level of 0.05

*CI* concentration index

We observed a negative association between FHC visits and disability status. The number of work/school days missed due to illness is negatively associated with FHC visits. People who had health problems over the past month reported more FHC utilization. We found that women age 65–74 were more likely to use FHC than the reference group. Educational level is negatively associated with FHC visits.

Individuals with disabilities tended to use more inpatient care at *soum* health centers.

There is a significant and positive association between a number of missed work/school days due to illness and inpatient care utilization. Respondents who had health problems over the past month were more likely to use inpatient care. Both women and men age 55 and older tended to use inpatient care in rural areas. Widowhood is positively associated with inpatient care. A negative association was found between being single/never married and inpatient care utilization. Individuals who were either self-employed or unemployed tended to use less inpatient care at *soum* health centers. Highly educated individuals were less likely to use inpatient care. Distance to a healthcare center was negatively associated with inpatient care utilization.

Table 3 also shows the results of the decomposition analysis, including each determinant’s concentration index and contribution percentage. Following is a plausible interpretation. Disability was concentrated among the poor in urban areas (concentration index is −0.195). It contributed −4.8% to the estimated degree of income-related inequality in FHC utilization.

If disability were equally distributed across income groups in urban areas or if there were no association between disability and FHC utilization, the estimated degree of income-related inequality in FHC utilization would be 4.8% higher.

Disability in rural areas was also concentrated among the poor (concentration index is −0.026). Its contribution to the estimated degree of income-related inequality in inpatient care utilization at *soum* health centers was −2.92%. If disability were equally distributed across income groups in rural areas or if there were no association between disability and inpatient utilization, the estimated degree of income-related inequality in inpatient care utilization at *soum* health centers would be 2.92% higher. Each of the other determinants can be interpreted in a similar fashion.

**Fig. 1:**
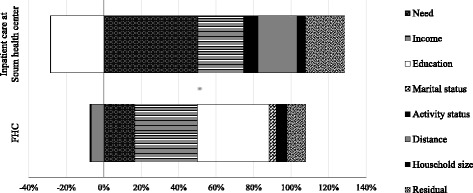
Decomposition analysis of income-related inequalities in FHC and inpatient care at *soum* health center in Mongolia, 2012

Figure 1 shows a summary of the contributions. In urban areas, income-related inequality in FHC utilization was caused mainly by income (33.25%), educational level (38.13%) and need distribution (16.75%). In rural areas, 50.2% of income-related inequality in inpatient care utilization at *soum* health centers was due to need distribution. Contributions of income (24.36%), distance to the nearest healthcare center (20.74%) and activity status (7.86%) were monitored. Educational level made a negative contribution (−28.32) to inequality, indicating that more highly educated people were less likely to use inpatient care at *soum* health centers.

## Discussion and conclusion

That sustaining universal health coverage is a fundamental step in tackling inequality in health is a well-known fact [26]. Well-designed and efficient PHC is crucial to moving toward universal health care [3].

Over the past decade, the Mongolian government has paid careful attention to ensuring a more equitable healthcare system, including affordable and high-quality services [5, 27]. PHC is now free and almost universally accessible, both in urban and rural settings, as a result of recent policy reforms. However, a lack of research that evaluates and assesses the current PHC model is limiting further improvement.

This study goes beyond previous research. First, it separately analyzes the degree of inequality in PHC services in urban and rural areas given the differing structures. Second, it tracks the degree of inequity in inpatient care utilization at *soum* health centers, the main providers of PHC in rural areas. Because of their important role, FGP/*soum* hospitals were restructured and renamed FHC/*soum* health centers in order to strengthen PHC services in accordance with the amendment to the health law in 2011 [5].

The study reveals a number of important findings. First, FHC utilization is concentrated among the poor in urban areas. This finding is consistent with results of the previous studies [15, 28]. However, direct comparisons are not feasible due to differences in study methods or healthcare utilization variables. For example, one study estimated inequality in outpatient utilization at the primary healthcare level using the concentration index, and others analyzed it at crude level regardless of the fact that PHC was provided differently in urban and rural areas [15, 28]. After controlling for need, the utilization of FHC still shows pro-poor equity. Consistent with this finding, international studies typically show that PHC utilization is concentrated among the poor regardless of differing payment methods and referral systems at the PHC [25, 29].

Higher utilization among the Mongolian poor may be due to government policies that favor the economically disadvantaged and vulnerable groups with the greatest health needs [27]. Previously, FHC was funded by social health insurance [5]. As a result, poor people who had emigrated from rural areas were not able to visit FHC because they were unregistered and uninsured. Since 2007, FHC is fully funded by the government budget, enabling those who are unregistered and uninsured to obtain PHC services. When it comes to the risk-adjusted capitation payment method, the rate for poor and vulnerable groups is higher than for the population as a whole. The method encourages FHCs to provide more health services to these groups.

On the other hand, this pro-poor inequality in FHC utilization could be a result of a deficient referral system. Reform of PHC aimed not only to provide universal access to quality health care, but addressed the financial sustainability of the system by introducing a gatekeeping strategy at the PHC [6]. However, those who are better off tend to go to the upper level hospitals regardless of their health needs or a fine for self-referral [5, 15]. These self-referrals may be linked to poor capacity, lower quality of care and work overload at FHC [11]. Those who suffered from catastrophic health payments were more likely to be the better off [16].

The results of the decomposition analysis indicate that pro-poor utilization in FHCs is caused mainly by income and educational level.

Second, our findings show that inpatient care utilization at *soum* health centers in rural areas was concentrated high among the rich. This indicates that there are still many barriers to health care in the remote areas on both the demand and supply side even though the government offers free inpatient and outpatient services at *soum* health centers. The results of the decomposition analysis show that income, economic activity (being herder and unemployed) and distance to the nearest healthcare center were the main contributors to pro-rich inequality in inpatient care utilization at *soum* health centers.

Some rural people have to travel more than 50 km to visit a center. This represents a significant financial burden on the poor. The ADB reported that *soum* health centers are generally clean and their services are managed well. Nonetheless, health centers could not serve wholly as primary health providers, given the poor transportation and communication infrastructure [6].

What’s more, the main sources of income in rural areas are herding and agriculture. Herders move every season in accordance with nomadic culture. As a result, they may choose a traditional healer or refrain from going to a health center if they do not have a serious condition.

Poor availability of health services contributes to the unequal distribution of health services in these areas. According to the MOH, there were 22.6 physicians and 33.1 nurses per 10,000 inhabitants in rural areas in 2015, 1.87 and 1.29 times lower than in Ulaanbaatar [30]. Most *soum* health centers lack laboratory equipment and pharmaceutical supplies [6].

Our results are inconsistent with the previous study. Bredenkamp et al. estimated inequality in inpatient care utilization at *soum* hospital/FGP based on HSES 2007/2008 data [28]. They found a statistically significant and negative concentration index, indicating that poor people tended to use more inpatient services at *soum* hospital/FGP. They applied the concentration index to their analysis, whereas we proceeded from the EI. The different choice of indices precluded comparisons.

Many positive changes have been documented in the Mongolian healthcare system since the PHC reform was adopted under the health sector development program funded by the ADB.

Based on our findings, however, we argue that the current PHC model is insufficient to sustain UHC and better health for everyone without a more forceful approach in both rural and urban areas. This has certain policy implications.

First, Mongolia should strengthen PHC, which means expanding capacity, improving quality, structuring the provision of services and ensuring more efficient funding. FHC capacity, including poor quality of health services and lack of skilled resources, is not meeting growing expectations [5], particularly among the better off [7]. Greater funding of FHC would improve the availability of health care, which might reduce self-referral to secondary or tertiary level hospital.

Second, it is necessary that primary health centers be the first level of contact and play a gatekeeping role. The system has lost its referral structure, reducing the function of PHC as a gatekeeper. As a result, high income individuals can bypass PHC and obtain outpatient and inpatient care at the upper level hospitals in both rural and urban areas. This not only widens the equality gap, but makes the health system more expensive and inefficient. Strong referral and gatekeeper strategies can structure demand more efficiently.

Third, ensuring free access to health services in remote areas does not guarantee equitable healthcare distribution unless transportation and communication infrastructure is improved. Such barriers will certainly be addressed over the long run. There is a need for more targeted policies that can be implemented in the short run to improve and expand access to health care in rural areas, specifically for the poor.

Fourth, and most importantly, income-related inequality in PHC utilization in rural and urban areas will not be eliminated unless immigration, poverty, unemployment and other social problems are addressed.

One limitation of the study is that the utilization of outpatient services at *soum* health centers was not covered in the paper since the result was insignificant. Further studies with a different living standard indicator may be called for. Additionally, inequality in primary health care use among the children can be explored in the further study, since the inclusion criterion in this study was that individuals be 18 or older.

## Abbreviations

ADB: Asian Development Bank
EI: Erreyger’s concentration indices
FGP: Family group practices
FHC: Family health centers
HI: Horizontal inequity
HSES: Household socio-economic survey
OLS: Ordinary least square
PHC: Primary health care
UHC: Universal health coverage
